# Light at the end of the tunnel?: The Great Indian Pharmacoeconomics story

**DOI:** 10.3389/fphar.2013.00153

**Published:** 2013-12-13

**Authors:** Karan B. Thakkar, Gauri Billa

**Affiliations:** Clinical Pharmacology and TherapeuticsMumbai, India

**Keywords:** pharmacoeconomics, drug costs, drug price control, inflation, out of pocket expenditure

It is estimated that 20 million people in India fall below the poverty line each year because of indebtedness due to healthcare needs (PricewaterhouseCoopers, 2007). This is indeed an alarming figure. Just to put things into perspective, the population of the financial capital of our country, Mumbai, is around 12 million and that of the national capital, Delhi, is 10 million. Now, imagine a population, the size of an entire city, going bankrupt. Only 11% of the Indian population have health insurance coverage (PricewaterhouseCoopers, 2007). Thus, everyone outside this 11% and those who don't fall in the upper middle and upper socio-economic classes, is at a risk of going bankrupt, God forbid, if they were to encounter a health crisis.

Total health care costs can be divided into two major components: cost of medicines and other costs. We would like to highlight the cost of medicines as a separate chunk because, the average Indian household may spend about 50% of their total health expenditures on medicines alone. According to the National Sample Survey (NSS) for the year 1999–2000, in rural India, the share of drugs in the total Out of Pocket expenditure (OPP) was estimated to be nearly 83%, while in urban India, it was 77% (NPPP, 2011). The other costs include doctor's fees, medical/surgical procedure costs, laboratory test costs, in-hospital costs and so on. And there are the intangible costs that are difficult to estimate but nevertheless assume significant proportions. It is important to note that most of the health insurance schemes pay the in-hospital costs only.

Inflation is touching a new high and the rupee is touching a new low. The cost of living is steadily increasing whereas the incomes of individuals are increasing at much lower rates. In India, the mighty “gold” and the acrid “onions” have been in the news for being one of the priciest commodities putting them out of the common man's reach. But drug prices are outstripping all commodities (Selvaraj, 2007). An examination of the price trends of 152 drugs in India, reveals that antibiotics, anti-tuberculosis and anti-malarial drugs, and drugs for cardiac disorders, etc. registered price increments from 1 to 15% per annum during 1976–2000 (National Commission on Macroeconomics and Health Ministry of Health and Family Welfare Government of India, 2005). The reason being that only one-tenth of drug market is price controlled as against nearly 90% during the late 1970s (Government of India, 2005).

If this was not enough, both communicable and lifestyle diseases are increasing at alarming proportions. It is estimated that by 2015 the number HIV/AIDS cases would be three times more than that in 2005, entailing possibly a corresponding increase in the existing prevalence level of tuberculosis of about 85,00,000 cases. Cardiovascular diseases and diabetes will more than double. Cancers will rise by 25%. In the coming 5 years there will be an enormous increase in various health disorders thereby increasing the healthcare costs geometrically (National Commission on Macroeconomics and Health Ministry of Health and Family Welfare Government of India, 2005).

At such times, the decision of the National Pharmaceutical Pricing Authority (NPPA), to regulate prices of 348-odd essential medicines by means of the Drugs Prices Control Order 2013, is a welcome respite (Department of Pharmaceuticals, 2013). But how long will this step go in mitigating the double blow of increasing disease and skyrocketing prices, only time will tell. Many loopholes have been cited in the DPCO (First Post – India, 2013; Rajagopal, 2013; Venkiteswaran, 2013). Some of them are as follows:

1. Only drugs on the National List of Essential Medicines (NLEM) will be included. The NLEM has itself been criticized for improper selection of drugs (Manikandan and Gitanjali, 2013).
2. Manufacturers might change dosages and formulations to avoid the DPCO.
3. “Market based pricing” has been used to determine the ceiling price.

The past experiences have not been good, because in 2008 the government had initiated a similar program called as the *“Jan Aushadhi”* program, that hit many roadblocks and was not successful at what it had purported (Jayaraman, 2010; Alexander, 2012). The private sector accounts for more than 80% of total health care spending in India (PricewaterhouseCoopers, 2007). Unless there is a decline in the combined central and state government deficit, which stands at roughly 8.5% as of 2012–13, the opportunity for significantly higher public health spending will also be limited (Government of India, 2013).

The government of India has started the *Rashtriya Swasthya Bima Yojana* (RSBY), literally “National Health Insurance Programme” for individuals below the poverty line in 2008. It provides annual hospitalization cover up to Rs. 30,000 or $ 485 for a family of five members through health insurance companies (Ministry of Labour, and Employment, 2013). But again it doesn't cover the outpatient costs. Also, it has been said that the coverage under the scheme is less than desirable and as a result many poor still remain uninsured (Dhoot, 2011, 2013). The exact impact of the scheme still remains to be evaluated (Dhoot, 2013). Another piece of irony is that since many villages do not even have hospitals, some of these hospitalization schemes can't be used at all (Sinha, 2012).

Pharmacoeconomics research can take us toward the final aim of making drugs affordable to all. Though pharmacoeconomics is in its infancy in India, it is rapidly evolving and good quality studies are being conducted across the nation. The first proposed Pharmacoeconomics guidelines draft was recently prepared by experts in the field (Gupta et al., 2013). This notwithstanding, overall, for the common man, the situation doesn't look very promising and a lot needs to be done to make medicines affordable to all.
